# Endoscopic ultrasound-guided pancreatic ring drainage via a pancreaticoenteric fistula

**DOI:** 10.1055/a-2638-3110

**Published:** 2025-07-29

**Authors:** Rishad Khan, Ryan Law

**Affiliations:** 1 6915 Division of Gastroenterology and Hepatology, Mayo Clinic, Rochester, Minnesota, United States

A 50-year-old patient with a history of necrotizing pancreatitis and disconnected pancreatic duct syndrome (DPDS) was referred for endoscopic management 1 . Cross-sectional imaging revealed a dilated main pancreatic duct (PD) with an abrupt cutoff at the genu ( Fig. 1 ).

**Fig. 1:**
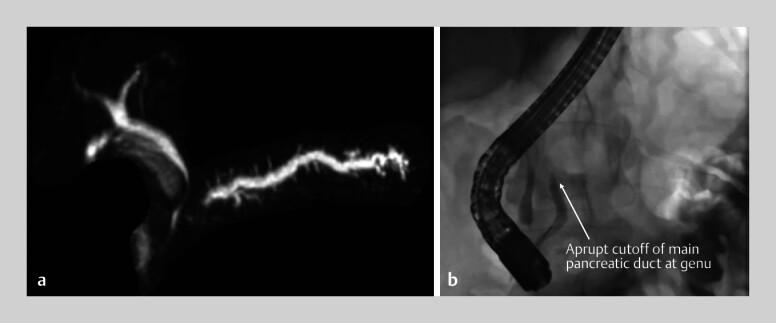
Magnetic resonance cholangiopancreatography (left) and endoscopic retrograde pancreatography (right) showing an abrupt cutoff of the main pancreatic duct at the genu.

During initial endoscopic retrograde pancreatography, a 0.018-in. guidewire and cannula could only be advanced into the ventral PD. A contrast pancreatogram revealed a cut-off at the genu. A 5-Fr by 3-cm pancreatic stent was placed for pancreatitis prophylaxis and as a marker for endoscopic ultrasound-guided PD drainage.

Initial transgastric endosonographic views showed the PD dilated to 5 mm, which was punctured with a 19-G needle ( Video 1 ). A contrast pancreatogram showed the duct in the body and tail and no PD opacification in the head. Pressurization of the PD with contrast showed a small fistulous tract communicating with the small bowel, likely sequelae from prior necrotizing pancreatitis ( Fig. 2 ). We utilized this fistulous tract to perform ring drainage 2 .

Endoscopic ultrasound-guided pancreatic ring drainage via a spontaneous pancreaticoenteric fistula for management of disconnected pancreatic duct syndrome.Video 1

**Fig. 2:**
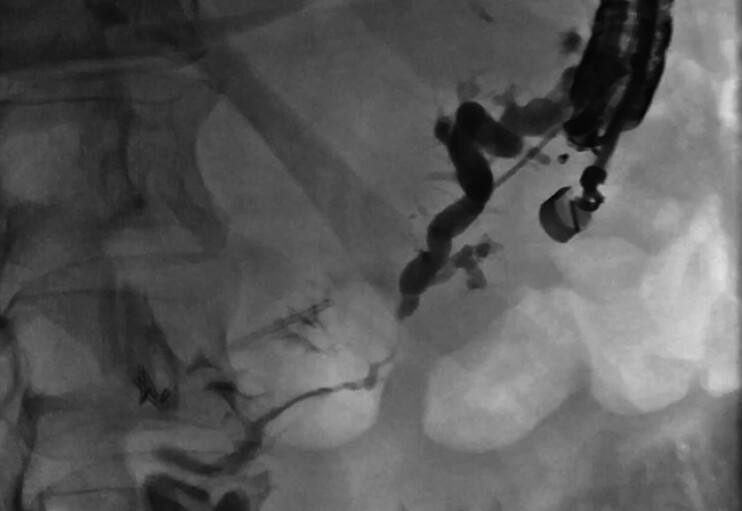
Contrast pancreatogram after EUS-guided pancreatic duct puncture showing a fistulous tract communicating with the small bowel.

A 0.025-in. guidewire was advanced into the PD, through the fistulous tract, and coiled in the small bowel. A 6-Fr cystotome was used to access the PD. This was followed by tract dilation with a 4- to 6-Fr rigid dilator and a 4-mm balloon dilator ( Fig. 3 ). One 7-Fr by 15-cm double-pigtail plastic stent was placed, with the distal pigtail deployed in the small bowel lumen and the proximal pigtail in the gastric lumen ( Fig. 4 ).

**Fig. 3:**
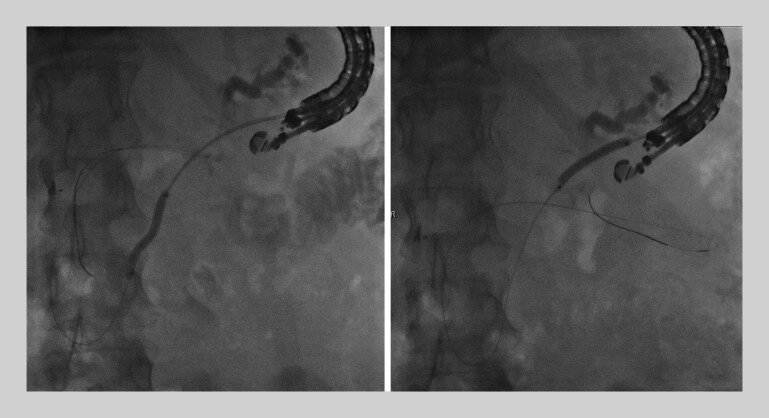
Dilation of the entire tract from the pancreaticoenteric fistula to the pancreaticogastrostomy.

**Fig. 4:**
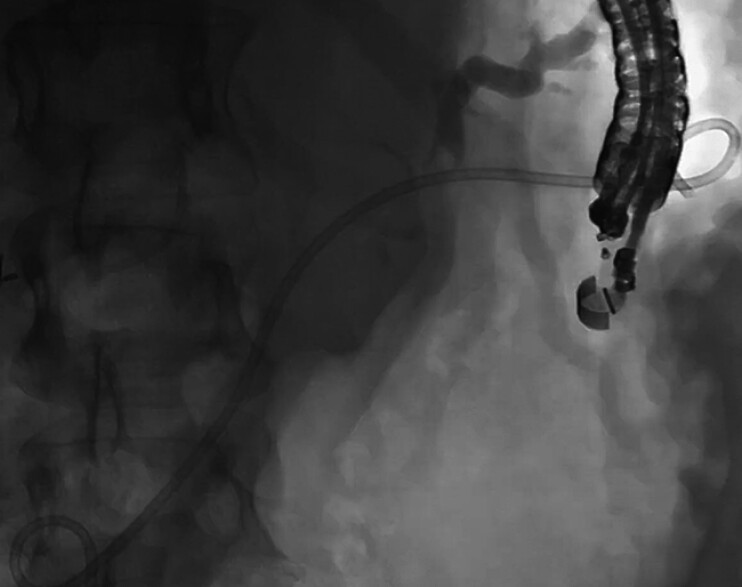
A 7-Fr by 15-cm double pigtail plastic stent, with the distal pigtail in the small bowel and the proximal pigtail in the gastric lumen.

At 13 months following her initial procedure, the patient’s recurrent episodes of pain have resolved. She continues to do well with regular stent exchanges twice yearly.

Endoscopic management of DPDS is often limited by an inability to pass a guidewire and any devices across the disconnected segment 3 . Here, we report a pragmatic approach whereby a spontaneous fistula was used to enable ring drainage with successful management of DPDS.

Endoscopy_UCTN_Code_TTT_1AS_2AI

## Notes

Endoscopy E-Videos https://eref.thieme.de/e-videos
*E-Videos* is an open access online section of the journal *Endoscopy* , reporting on interesting cases and new techniques in gastroenterological endoscopy. All papers include a high-quality video and are published with a Creative Commons CC-BY license. Endoscopy E-Videos qualify for HINARI discounts and waivers and eligibility is automatically checked during the submission process. We grant 100% waivers to articles whose corresponding authors are based in Group A countries and 50% waivers to those who are based in Group B countries as classified by Research4Life (see: https://www.research4life.org/access/eligibility/ ). This section has its own submission website at https://mc.manuscriptcentral.com/e-videos .
